# Many-to-one comparisons after safety selection in multi-arm clinical trials

**DOI:** 10.1371/journal.pone.0180131

**Published:** 2017-06-26

**Authors:** Gerald Hlavin, Lisa V. Hampson, Franz Koenig

**Affiliations:** 1 Section for Medical Statistics, Center for Medical Statistics, Informatics, and Intelligent Systems, Medical University of Vienna, Vienna, Austria; 2 Statistical Innovation, Advanced Analytics Centre, AstraZeneca, Cambridge, United Kingdom; 3 Department of Mathematics & Statistics, Lancaster University, Lancaster, United Kingdom; New Jersey Institute of Technology, UNITED STATES

## Abstract

In phase II platform trials, ‘many-to-one’ comparisons are performed when *K* experimental treatments are compared with a common control to identify the most promising treatment(s) to be selected for Phase III trials. However, when sample sizes are limited, such as when the disease of interest is rare, only a single Phase II/III trial addressing both treatment selection and confirmatory efficacy testing may be feasible. In this paper, we suggest a two-step safety selection and testing procedure for such seamless trials. At the end of the study, treatments are first screened on the basis of safety, and those deemed to be sufficiently safe are then taken forwards for efficacy testing against a common control. All safety and efficacy evaluations are therefore performed at the end of the study, when for each patient all safety and efficacy data are available. If confirmatory conclusions are to be drawn from the trial, strict control of the family-wise error rate (FWER) is essential. However, to avoid unnecessary losses in power, no type I error rate should be “wasted” on comparisons which are no longer of interest because treatments have been dropped due to safety concerns. We investigate the impact on power and FWER control of multiplicity adjustments which correct efficacy tests only for the number of safe selected treatments instead of adjusting for all *K* null hypotheses the trial begins testing. We derive conditions under which strict control of the FWER can be achieved. Procedures using the estimated association between safety and efficacy outcomes are developed for the case when the correlation between endpoints is unknown. The operating characteristics of the proposed procedures are assessed via simulation.

## 1 Introduction

Clinical trials are often complex and costly undertakings, and many patients may be required to reach a conclusion on whether a new therapy is efficacious. One way to reduce the logistical burden of drug development is to simultaneously study *K* novel treatments within a single multi-arm trial which also includes a common control arm. Comparing treatments with the same control group can yield substantial reductions in sample size because only one control is needed compared with the *K* control groups that would be needed by *K* independent two-arm trials [1]. Such Phase II platform trials are particularly appealing as an efficient way to identify effective treatments for small populations, when reasonable numbers of patients can be challenging to recruit [2, 3]. In principle, multi-arm trials are appropriate for evaluating not only different treatments but also different patient subgroups, doses, etc.

Once promising treatments have been identified, they are then traditionally taken forwards to separate, confirmatory, Phase III trials. In this paper, we consider the design of a single-stage Phase II/III trial intended to address both treatment selection *and* confirmatory efficacy testing. Traditionally, confirmatory trials must ensure the family-wise error rate (*FWER*), defined as the probability of claiming efficacy for at least one ineffective treatment, is controlled at a suitably small level, which we denote by *α*_*nom*_ [4] (for example, *α*_*nom*_ = 0.05 or *α*_*nom*_ = 0.025 for two-sided and one-sided tests, respectively), for every configuration of ineffective treatments. The chance of making a false claim of efficacy for at least one treatment will increase as *K* increases if the null hypothesis associated with each experimental treatment is naively tested at local level *α*_*nom*_ [5]. Multiple comparison procedures are regularly applied to correct the local significance level at which each hypothesis test is performed to ensure that strong control of the *FWER* level is maintained. When the primary efficacy endpoint is continuous, a standard parametric procedure which accounts for the correlation structure induced by the common control is the Dunnett test [6]. Under this and other methods (see, for example, [7]), the reduction in the local significance level becomes more severe as the number of comparisons increases [8–10].

Safety is of paramount importance in all phases of drug development. Suppose that at the end of the Phase II/III trial, treatments may be abandoned due to safety concerns before being tested for efficacy. Should one or more treatment arms be dropped in this way, when testing the efficacy of the remaining treatments relative to control it would seem natural to perform multiplicity corrections based only on the number of remaining treatments. Intuitively, it seems unreasonable to spend parts of the overall nominal significance level on treatments for which null hypotheses are not tested.

We will demonstrate that, in general, such a procedure (which selects safe treatments in a data-driven way and then performs a multiplicity correction based on the remaining treatments) will not control the *FWER*. However, we state sufficient conditions under which this procedure does control the *FWER*; this will provide reassurance to clinical trialists should they adopt designs satisfying these criteria. Furthermore, we propose a testing procedure which maintains adequate FWER control should the sufficient conditions fail to hold. Throughout this manuscript we restrict attention to the case where toxicity and efficacy are both measured by normally distributed variables (where lower toxicity but higher efficacy measurements are more favourable), a framework already used in similar settings [11–13].

Continuous safety parameters are often closely connected to the treatment of a patient. For example spironolactones that are used in the treatment of patients with high blood pressure with spironolactones, attention has to be paid on blood potassium levels, where increased levels can lead to abnormal heart rhythms and in severe cases to cardiac arrests [14]. Another example concerns treatments with agomelatine, a melatonergic antidepressants. Raised levels of transaminases which indicate liver damage, are a contraindication and a prespecified reason to discontinue a treatment using Valdoxan (a trade name of agomelatine) [15]. Another relevant continuous safety parameter in drug development is the assessment of the QT-time, a time interval in the electrical cycle of the heart [16]. A QT-time prolongation is especially relevant in psychoactive drugs [17]. In all of the aforementioned examples, certain continuous parameters are important to judge the safety and therefore the usefulness of a medicine.

On the other hand multi arm trials are increasingly demanded in modern clinical research [1–3]. In [18] an extensive list on multi arm trials published in 2009 is included, where 221 trials with 3 or more arms reported to have a parallel group design. Another example of a multi arm trial is [19], a study of Eplerenone (a selective aldosterone blocker intended for treatment of hypertension), where 417 patients were randomized to 6 Eplerone arms, 1 Spironolactone arm, and placebo. There the comparisons to placebo (or aldosterone) in serum aldosterion levels, total plasma renin, and active plasma renin were done using the Dunnett test. As an important tolerability endpoint, mean changes in blood potassium levels were measured. A further example is a study of mixed amphetamine salts with extended releae for the treatment of Attention-Deficit/Hyperactivity Disorder (ADHD) [20]. Efficacy comparisons of 3 treatment arms to placebo were again done using the Dunnett test. Besides adverse events, safety analysis included the measurements several continuous safety variable, e.g. mean colesterol decreases and ECG measurements like the aforementioned QT-time.

These examples show the potential of uses of our two-step approach: the structured combination of multi-arm trials with safety considerations. In S1 Example, a numerical example will illustrate this approach.

In Section 2, we present a motivating example which illustrates the testing problem at hand and introduces the terminology used throughout this article. In Section 3, general notation is defined and the bivariate normal data model is presented. The concept of maximum *FWER* inflation is explained in Section 4. There we also present simulation results which show how maximum FWERs vary with the correlation between normally distributed toxicity and efficacy measurements, to see what can go wrong by performing safety selection before efficacy testing. For the procedure basing multiplicity corrections on the number of selected treatments, it is proven that the maximum *FWER* inflation is monotonically increasing for decreasing correlations, and therefore the maximum occurs when the correlation is −1. Then the main result of this paper is presented, which states that so long as toxicity and efficacy have a non-negative correlation, safety selection can be applied without inflating the *FWER*. The proof of this result can be found in the Appendix. In Section 5, adjustments are proposed for the case when a positive (or zero) correlation cannot be assumed. A simulation study in Section 6 compares different methods with a conservative procedure which always corrects the local signficance levels of hypothesis tests for all *K* pre-specified comparisons.

## 2 Motivating example

Suppose we have three treatment groups indexed by *i* ∈ {1, 2, 3} with corresponding expected efficacy outcomes *μ*_1_, *μ*_2_, and *μ*_3_, which we wish to compare with a common control (indexed by *i* = 0) on which the mean efficacy outcome is *μ*_0_. Treatment groups may represent, for example, different treatment regimens, drugs, or drug levels. For *i* ∈ {1, 2, 3}, the elementary null-hypothesis *H*_*i*_: *μ*_*i*_ − *μ*_0_ ≤ 0 which states that treatment *i* is not more efficacious than control, will be tested against the alternative $H_{i}^{A}:\mu_{i}-\mu_{0}>0$. We reject *H*_*i*_ if the corresponding test statistic exceeds a pre-specified threshold denoted by *b*_*i*_. Let us assume that the FWER is to be controlled at a pre-specified level *α*_nom_. Then, the decision thresholds *b*_1_, *b*_2_, and *b*_3_ have to be chosen such that under any configuration *J* ⊆ {1, 2, 3} of true null-hypotheses (indexing treatments which are not efficacious relative to control), the probability of rejecting at least one true null-hypothesis is less or equal to *α*_nom_. For ease of presentation, suppose that to achieve FWER control we apply the simple Bonferroni correction. Then we split the nominal level *α*_nom_ equally among all elementary null-hypotheses *H*_*i*_, i.e., each *H*_*i*_ is tested separately at local significance level *α*_nom_/3. In subsequent sections, we will consider the Dunnett test to correct for multiple hypothesis testing; however, even under this approach, the arguments presented in this example remain essentially unchanged.

Safety, as well as efficacy, is an important issue in clinical drug development. If a treatment appears to be unacceptable on the basis of observed toxicity data, should it still be considered for efficacy testing? If the answer is no, then the next question is how can we select on the basis of safety and still control the FWER for efficacy testing? To address this, let us continue with our example and suppose that at the end of the trial, treatment 3 is considered unsafe. Then it would seem unnatural to test the remaining hypotheses *H*_1_ and *H*_2_ at local significance level *α*_nom_/3, since this wastes 1/3 of *α*_nom_ on a treatment comparison that is no longer of interest. Although a Bonferroni correction based on the initial number of planned treatment-control comparisons would be valid in terms of FWER control, regardless of how treatments are selected, it may result in a conservative procedure. A more natural and less conservative approach would be to correct for the number of treatment-control comparisons that are actually performed after safety selection; in our example, this corresponds to testing *H*_1_ and *H*_2_ at a multiplicity-adjusted level of *α*_nom_/2. We will refer to the former correction for initially planned treatment-control comparisons as the ‘*conservative correction*’ and the latter approach as the ‘*natural correction*’.

For the remainder of this paper, we will formally distinguish between the *safety selection step*, where unsafe treatments are dropped, and the *efficacy testing step*, where the remaining sufficiently safe treatments are each compared with a common control. The *overall two-step procedure* is then the combination of both steps. Here it is implied, that for each selection of treatments a multiple testing procedure is defined for this selection.

First focusing on such a multiple testing procedure in the efficacy testing step for treatments defined by the index set *S* ⊆ {1, 2, 3}, let *FWER*(*S*, *J*) denote its *unconditional* (i.e. not conditioning on any safety data and thus on how *S* was derived) probability for rejecting at least one of the hypotheses *H*_*i*_, *i* ∈ *S* ∩ *J*. Here *J* again denotes the set of true null-hypotheses as defined above. We say that for the efficacy testing step, FWER control is maintained at level *α*_nom_ if *FWER*(*S*) := max_*J* ⊆ {1, 2, 3}_ *FWER*(*S*, *J*) ≤ *α*_nom_ for all possible subsets *S*. Given a predefined safety selection rule, *FWER*_*o*_ denotes the *FWER* of the overall two-step selection and testing procedure. We say that *FWER* control for the overall two-step procedure is maintained at level *α*_nom_ if *FWER*_*o*_ ≤ *α*_nom_.

In our example, testing each null hypothesis indexed by *S* ⊆ {1, 2, 3} at significance level *α*_nom_/|*S*| (here | ⋅ | denotes the cardinality of *S*) leads to *FWER*(*S*) ≤ *α*_nom_, thus *FWER* is controlled in the efficacy testing step at level *α*_nom_. However for particularly structured data this does not imply *FWER*_*o*_ control. The intuition here is, that if the selection of a treatment for being tested for efficacy is associated with increased efficacy observations, *FWER*_*o*_ can be increased. This becomes particularly obvious, if for example an (unreasonable) selection procedure would always select just the most promising treatment in terms of efficacy and therefore no correction of the significance level is performed. In contrast, *FWER* control in the efficacy testing step implies *FWER*_*o*_ control for the conservative correction (where null hypotheses for selected treatments are tested at level *α*_nom_/3) because rejection regions in this case do not depend on which and how many other treatments are selected. Therefore the inclusion of a selection procedure that precedes efficacy testing, can only reduce the number of false positive findings.

The main focus of this manuscript is on defining conditions under which the natural correction in the efficacy testing step leads to *FWER*_*o*_ control, and on determining how to adjust this correction to ensure *FWER*_*o*_ control when these conditions do not hold.

## 3 Model specification

As a general framework, we begin by assuming efficacy and toxicity data are normally distributed.

### 3.1 General framework

Let *I* := {1, …, *K*} be the set of all experimental treatments. Denoting the average efficacy outcome in group *i* as *μ*_*i*_, *H*_*i*_: *μ*_*i*_ − *μ*_0_ ≤ 0, *i* ∈ *I* defines the elementary hypothesis stating that treatment *i* cannot be regarded as more efficacious than the control (treatment 0).

We define the indicator *τ*_*i*_, where *τ*_*i*_ = 1 when treatment *i* is excluded from efficacy testing on the basis of the observed toxicity data, whereas *τ*_*i*_ = 0 indicates that treatment *i* is selected for efficacy testing. Then

$$\begin{array}{c} \varsigma_{S}:=\prod_{i\in S}\left(1-\tau_{i}\right)\cdot \prod_{j\in I\setminus S}\tau_{j} \end{array}$$

indicates the event that exactly the treatments in the (sub-)set *S* ⊆ *I* are selected for efficacy testing, whereas the remaining treatments in *I* ∖ *S* are dropped due to safety concerns.

At the end of the two-stage procedure, we represent the test decision on the rejection of an elementary null hypothesis *H*_*i*_ by a binary indicator. As we have seen from our motivating example, the final decision rule for a treatment will depend on the safety selection step. For instance, the natural multiplicity correction depends on the actual number of treatments selected for efficacy testing, that is, |*S*|. For each selection set *S* ∈ *I*, let $\eta_{i}^{S}$ for *i* ∈ *S*, denote a binary indicator, where $\eta_{i}^{S}=1$ denotes rejection of the *i*th null hypothesis, otherwise $\eta_{i}^{S}=0$. Here the definition of $\eta_{i}^{S}$ depends on the selection subset, which is reflected by the superscript *S*. Since unsafe treatments are not subject to efficacy testing, we set $\eta_{i}^{S}=0$ for *i* ∈ *I* ∖ *S*, without performing a statistical test of *H*_*i*_.

Since *J* ⊆ *I* denotes the set of all true null-hypotheses,

$$\begin{array}{c} \varphi_{J}^{S}:=1-\prod_{i\in S\cap J}(1-\eta_{i}^{S})\; \end{array}$$

indicates a type I error has been committed for one of the selected but inefficacious treatments. The *FWER* is controlled in the efficacy testing step at level *α*_nom_ if, for each possible selection set *S* ⊆ *I* and each configuration of true null-hypotheses *J*, it holds that $E[\varphi_{J}^{S}]=FWER(S)\leq \alpha_{\text{nom}}$, where *FWER*(*S*) := max_*J*⊆*I*_ *FWER*(*S*, *J*). For the overall two-step procedure, *FWER*_*o*_ is controlled at the nominal level if

$$\begin{array}{c} \sum_{S\in M_{J}}E[\varsigma_{S}\cdot \varphi_{J}^{S}]\leq \alpha_{\text{nom}}. \end{array}$$ (1)

Here $M_{J}:=\left\{S\subseteq I:S\cap J\neq \emptyset \right\}$ contains all possible selections with at least one true null hypothesis.

If *ς*_S_ and $\varphi_{J}^{S}$ are independent, then the addends in Eq (1) would decompose to $E[\varsigma_{S}\cdot \varphi_{J}^{S}]=E[\varsigma_{S}]E[\varphi_{J}^{S}]$. In this case, *FWER* control in the efficacy testing step at level *α*_nom_ implies *FWER*_*o*_ control at the same level, since the sum of the $E[\varsigma_{S}]$ is less than or equal to one and therefore Eq (1) holds. In general we have

$$\begin{array}{ll} E\left[\varsigma_{S}\cdot \varphi_{J}^{S}\right] & =P\left(\varsigma_{S}=1,\varphi_{J}^{S}=1\right)\\ & =P\left(\varsigma_{S}=1|\varphi_{J}^{S}=1\right)\cdot P\left(\varphi_{J}^{S}=1\right)\\ & =E\left[\varsigma_{S}|\varphi_{J}^{S}=1\right]E\left[\varphi_{J}^{S}\right], \end{array}$$

but the $E[\varsigma_{S}|\varphi_{J}^{S}=1]$ do not necessarily sum to a value less than or equal to one.

### 3.2 The bivariate normal model

For the control group (*i* = 0), all treatment groups *i* ∈ *I*, and all patients *j* ∈ {1, …, *n*_*i*_}, we consider a two dimensional vector of measurements (*x*_*ij*_, *y*_*ij*_) coming from a bivariate normal distribution *N*((*μ*_*i*_, *θ*_*i*_), **Σ**) with $\Sigma =\left[\begin{array}{cc} \sigma_{i}^{2} & \rho_{i}\sigma_{i}\omega_{i}\\ \rho_{i}\sigma_{i}\omega_{i} & \omega_{i}^{2} \end{array}\right]$. Here *x*_*ij*_ and *y*_*ij*_ denote an efficacy and a toxicity measurement, respectively. Higher values for *x*_*ij*_ and *y*_*ij*_ shall be interpreted as higher efficacy and toxicity, respectively. Pairs of outcomes (*x*_*ij*_, *y*_*ij*_) are assumed to be independent across values of *i* and *j*. In what follows, let **x**_*i*_ and **y**_*i*_ denote the *n*_*i*_-dimensional vectors of efficacy and toxicity outcomes measured in group *i*, respectively.

For the safety selection stage, $\tau_{i}:=1_{\left\{(b_{i}^{y},\infty )\right\}}(T_{i}^{y}(y_{i}))$ is defined before the start of the trial. Here $T_{i}^{y}$ is a pre-specified function of the group *i* toxicity data which is compared with the pre-defined threshold $b_{i}^{y}$ (here **1** denotes the indicator function, so that *τ*_*i*_ = 1 when $b_{i}^{y}<T_{i}^{y}(y_{i})$ and 0 otherwise).

For the purposes of efficacy testing, data are summarised by the test statistics $T_{i}^{x}$ and the decision for null hypothesis *H*_*i*_ is given by $\eta_{i}^{S}:=1_{\left\{(b_{i,S}^{x},\infty )\right\}}(T_{i}^{x}(S,D))$ with pre-defined thresholds $b_{i,S}^{x}$ and *D* := {**x**_0_, …,**x**_*K*_}. In our framework these critical thresholds are chosen such that the *FWER*(*S*) is controlled (and therefore depend on the number of treatments in the selected set *S*).

In what follows, when determining the efficacy success criteria we will focus on an important multiple testing procedure in the many-to-one comparisons framework of normally distributed variables, namely the Dunnett Test. We will consider the case of equal group variances (an assumption that will be discussed in Remark 2 in the Appendix), that is $\sigma_{i}^{2}=\sigma^{2}\;\forall i\subseteq I\cup \left\{0\right\}$, for both the Dunnett test for unknown (DT) and known variances (ZT), where in the latter case hypothesis test decisions are based on Z-statistics. For both DT and ZT, *FWER*(*S*) is easily determined, since on the one hand the dependence on *S* is only due to the number of treatments in *S*, on the other hand in these cases the simplification of above definition *FWER*(*S*) = *FWER*(*S*, *I*) = *FWER*(*S*, *S*) holds. The straightforward definition of DTs or ZTs for every subset *S* by using |*S*| therefore ensures FWER control in the efficacy testing step. Similar arguments apply also for the Bonferroni correction.

### 3.3 Example (Cont.)

We continue the motivating example in Section 2, but assume a Dunnett test (with known variance) at one-sided level *α*_nom_ = 0.025) instead of a Bonferroni correction to correct for multiple comparisons.

In our notation the selection of treatments 1 and 2 would be implied by observing $T_{i}^{y}(y_{i})\leq b_{i}^{y}$ for *i* ∈ {1, 2} and $b_{j}^{y}<T_{j}^{y}(y_{j})$ for *j* = 3. Applying the natural correction in the efficacy testing step would now require the Dunnett test for two treatments. Assuming for example a variance of *σ*_*i*_ = *σ* = 1 and a group sample size of *n*_*i*_ = 22 for *i* ∈ {0, 1, 2} requires boundaries of $b_{1}^{x}=b_{2}^{x}=2.21$. In terms of p-values this would correspond to *α*-levels *α*_1_ = *α*_2_ = 0.0135. In comparison the conservative procedure would still use the boundaries $b_{1}^{x}=b_{2}^{x}=2.35$ (corresponding to *α*_1_ = *α*_2_ = 0.0094), which are adequate for a Dunnett test with three treatment-control comparisons

## 4 FWER of the two-step procedure

In this section the relationship between the within patient correlation of safety and efficacy measurements and the *FWER*_*o*_ is investigated. First, simulation results will be presented for the case of comparing two treatments groups with a single control and the maximum *FWER*_*o*_ will be calculated for different correlations. Then we will present sufficient conditions which ensure *FWER*_*o*_ control of the overall two-step procedure.

### 4.1 FWER maximizing correlation

We will show that *FWER*_*o*_ control does not necessarily hold if the natural correction is applied to negatively correlated toxicity and efficacy data. In our scenario, the nominal one-sided significance level was fixed at *α*_nom_ = 0.025. We will consider comparisons of *K* treatment groups with a common control assuming patient responses follow the bivariate normal model defined in Section 3, setting $\sigma_{i}^{2}=1$, for *i* = 0, 1, …, *K*, and $\omega_{i}^{2}=1$, for *i* = 1, …, *K*, without loss of generality. For simplicity, we also assume equal correlations *ρ*_1_ = … = *ρ*_*K*_ = *ρ*. Basing efficacy decisions on one-sided two-sample z-tests, to find a clinically relevant effect of *μ*_*d*_ = 1 with power 1 − *β* = 0.9, the per-group sample size is set to $n=2{\left(z_{1-\alpha }+z_{1-\beta }\right)}^{2}/\mu_{d}^{2}$.

Similar to [12], we calculate the worst case *FWER*_*o*_ of our test procedure for the situation when all expected responses are zero, that is, when *μ*_*i*_ = 0, for *i* = 0, … ,*K*. This is the situation where for the classical Dunnett test the FWER is maximized. Furthermore we set *θ*_*i*_ = 0, for *i* = 1, …, *K*. We then search across values of the toxicity thresholds $b_{1}^{y},\ldots ,b_{K}^{y}$ for monitoring test statistics $T_{i}^{y}\left(y_{i}\right):={\bar{y}}_{i}$ to find the configuration at which the maximum (worst case) *FWER*_*o*_.

**Fig 1:**
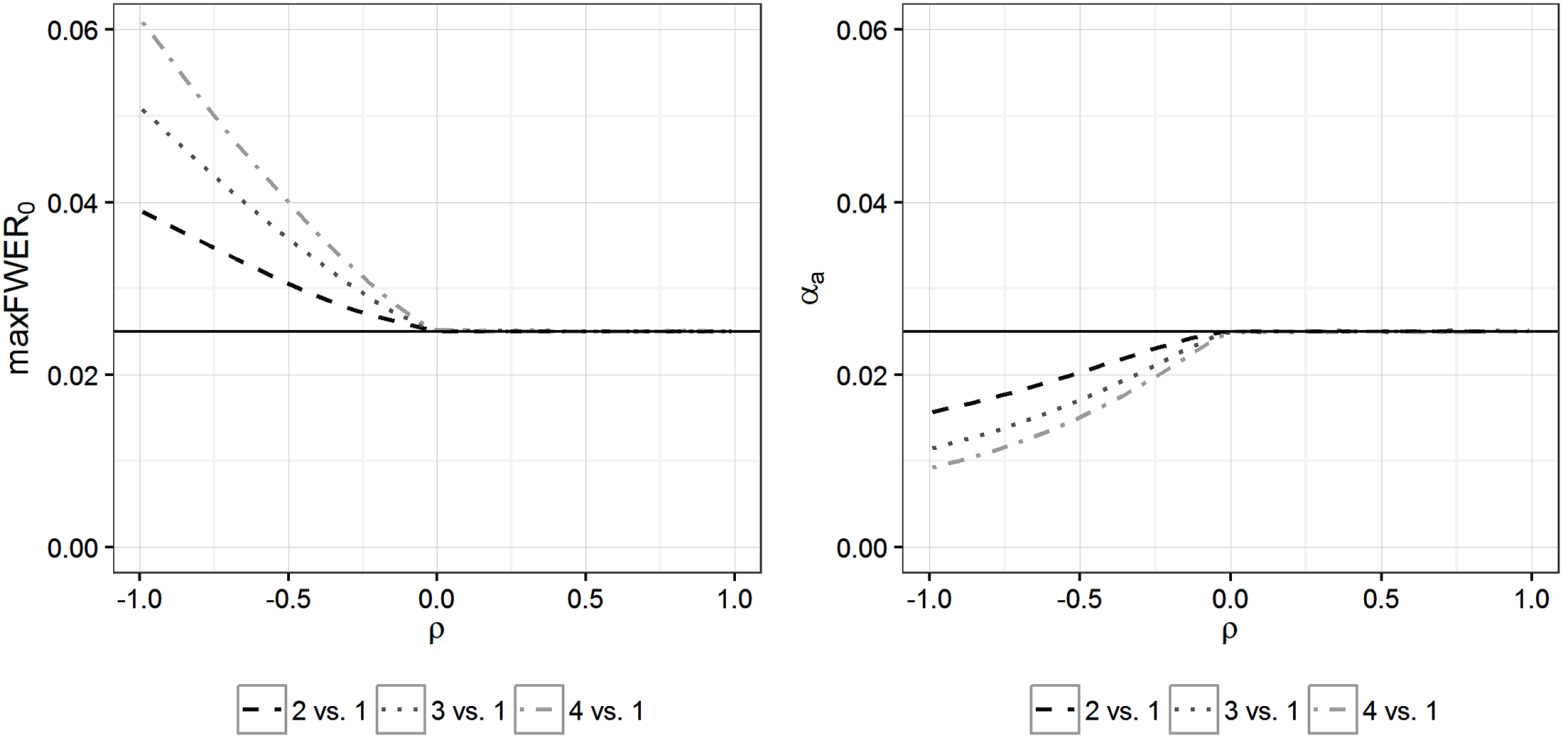
Maximum *FWER*_*o*_ (left) and corresponding adjusted nominal levels (right). In the left figure, worst case *FWER*_*o*_ for varying correlations *ρ* between toxicity and efficacy are drawn for the ZT for 2, 3, and 4 treatments. The maximum *FWER*_*o*_ is constant for values of *ρ* ≥ 0 and increases for decreasing *ρ* < 0. At *ρ* = −1 the maximum worst case *FWER*_*o*_ is reached. In the right figure, the corresponding adjusted nominal levels *α*_*a*_ to be used in a ZT correcting for the number of selected treatments are presented as a function of the true group correlations *ρ* (as described in Section 5.1). Positive correlation implies *FWER*_*o*_ control, in which case *α*_*a*_ = *α*_nom_. In case of negative correlation the *FWER* in the efficacy testing step has to be controlled at a specific level *α*_*a*_ < *α*_nom_ to ensure control of the worst case *FWER*_*o*_ at level *α*_nom_.

In Fig 1 (left), values of *FWER*_*o*_ maximized for all toxicity parameter configurations and thresholds are shown for different numbers of treatments *K* ∈ {2, 3, 4} and different correlations *ρ* ∈ (−1, 1) in the ZT situation (where the variances are known). The worst case *FWER*_*o*_ is maximized at *ρ* = −1 and decreases for increasing correlation until *ρ* = 0 is reached. For non-negative correlation, the worst case *FWER*_*o*_ is constant at the nominal level *α*_nom_.

Fig 1 (left) suggests that the worst case *FWER*_*o*_ inflation is maximized at *ρ* = −1. The following theorem provides a general statement about this observation. Here it is again assumed that $T_{i}^{y}={\bar{y}}_{i}$, but similar results are also straightforward to prove so long as after transformation of the data, a multivariate normal distribution is the resulting model.

**Theorem 1**. *Consider the bivariate normal model in* Eq (3.1) *and let* ∅ ≠ *J* ⊆ *I* *be the set indexing inefficacious treatments. Let safety selection be based on the group mean toxicities* ${\bar{y}}_{i}$. *Then the worst case FWER_o_ for the ZT and the DT is reached at ρ_i_* = −1, ∀*i* ∈ {1, …, *K*}.

A proof of this result can be found in the Appendix.

### 4.2 FWER control for positive correlation

Fig 1 (left) indicates that a worst case *FWER*_*o*_ inflation will not occur if the correlation between toxicity and efficacy responses is non-negative. Using the concept of association introduced in [21], we can generalize this observation with the following theorem:

**Theorem 2**. *Consider the bivariate normal model in* Eq (3.1) *and let* ∅ ≠ *J* ⊆ *I* *be the set indexing inefficacious treatments. Let safety selection be based on the group mean toxicities* ${\bar{y}}_{i}$. *If ρ_i_* ≥ 0, ∀*i* ∈ *I*, *and FWER(S)* ≤ *α_nom_* *holds* ∀*S* ⊆ *I*, *then the FWER of the overall procedure is also controlled at level α_nom_ for the ZT and the DT*.

The proof of Theorem 2 is presented in the Appendix, together with a discussion of possibilities and limitations of extensions.

## 5 Correlation-based adjustments

We have seen that when applying the natural correction, the *FWER*_*o*_ depends on the true correlation *ρ*_*i*_. For known non-negative correlation it is reasonable to prefer the natural to the conservative correction, since the former approach achieves higher power, while the *FWER*_*o*_ is still controlled. To compare *FWER*_*o*_ for different procedures we write $FWER_{o}^{\text{NA}}(\rho )$ when using the natural correction and when the true correlation is *ρ* (NA stands for natural correction). Here the assumption is equality of correlations across all treatment groups or, if equality cannot be assumed, we substitute the minimum correlation between toxicity and efficacy found across all groups. Using the monotonicity of the worst case *FWER*_*o*_ as a function of the true correlation as discussed in the context of Theorem 1, this choice of *ρ* then serves to derive an upper bound for the $\text{max}FWER_{o}^{NA}$. For the conservative procedure, we write $FWER_{o}^{\text{CO}}$, where CO stands for *conservative*. It is clear that $\text{max}FWER_{o}^{\text{NA}}(\rho )\geq \text{max}FWER_{o}^{\text{CO}}$ for all correlations *ρ*.

In this section our goal is to define an adjustment to the natural multiplicity correction for *known negative* correlation which can be used as an alternative to the conservative test procedure. Then we apply this idea to case of *unknown* correlation.

### 5.1 Known correlation

We have seen that $\text{max}FWER_{o}^{\text{NA}}(\rho )>\alpha_{\text{nom}}$ for *ρ* < 0. To avoid any inflation in the family-wise error rate, our proposed strategy is to apply the natural correction to a nominal significance level *α*_*a*_ ≤ *α*_nom_ which is chosen so that *FWER*(*S*) ≤ *α*_*a*_, for all subsets *S* ⊆ *I*, implies *FWER*_*o*_ ≤ *α*_nom_. This means that at the efficacy testing stage, each test statistic relating to an elementary null hypothesis for a selected treatment is compared with the Dunnett thresholds calculated at nominal level *α*_*a*_ adjusting for the number of selected treatments.

In Fig 1 (right), values of *α*_*a*_ used in the adjustment of the natural correction are drawn for different true and equal correlations in the ZT framework when comparing 2, 3, and 4 treatments with a common control. Of course these curves behave “inversely” to the curves shown in Fig 1 (left). Since for *ρ* ≥ 0, no *FWER*_*o*_ inflation occurs and therefore no adjustment of the nominal level is necessary we have *α*_*a*_ = *α*_nom_. In other words, in case of non-negative correlation, the correlation-adjusted two-step procedure is identical to the natural procedure, because due to Theorem 2 the latter controls the *FWER*_*o*_. For negative values of *ρ* however, to decrease the maximum *FWER*_*o*_ from $FWER_{o}^{\text{NA}}(\rho )>\alpha_{\text{nom}}$ to $FWER_{o}^{\text{KC}}(\rho )\leq \alpha_{\text{nom}}$, the natural correction has to be applied based on a lower significance level *α*_*a*_ < *α*_nom_ (KC means *known correlation*). The lowest value of *α*_*a*_ has to be applied at *ρ* = −1, where the worst case $FWER_{o}^{\text{NA}}$ is maximized.

### 5.2 Unknown correlation

In this section we extend our designs to accommodate an unknown correlation. If, in the method described above, the adjusted nominal level, *α*_*a*_, is calculated assuming the common correlation is *ρ*_*a*_ when in fact *ρ* is the true correlation, we write $FWER_{o}^{\text{KC}}(\rho ,\rho_{a})$ for the *FWER*_*o*_. In this more general notation, we can write $FWER_{o}^{\text{KC}}(\rho )=FWER_{o}^{\text{KC}}(\rho ,\rho )$ and $FWER_{o}^{\text{NA}}(\rho )=FWER_{o}^{\text{KC}}(\rho ,0)$. Our results so far indicate that *ρ*_*a*_ < *ρ* < 0 implies conservatism, that is, $\text{max}FWER_{o}^{\text{KC}}(\rho ,\rho_{a})<\alpha_{nom}$, which implies that our adjustment is more severe than necessary. The opposite is true when *ρ* < *ρ*_*a*_ < 0, in which case $\text{max}FWER_{o}^{\text{KC}}(\rho ,\rho_{a})>\alpha_{nom}$.

We propose basing multiplicity adjustments on an estimate $\hat{\rho }$ of the common correlation. To account for the variability of the estimate we define $FWER_{o}^{\text{PI}}:=E[FWER_{o}^{\text{KC}}(\rho ,\hat{\rho })]$, where the expectation is taken over realizations of $\hat{\rho }$ (PI stands for ‘*plug-in*’).

The standard way to define $\hat{\rho }$ is as follows:

1. Estimate the empirical correlation coefficients *r*_*i*_ between efficacy and toxicity in group *i*, ∀*i* ∈ *I*.
2. Apply Fishers’s z-transformation (see e.g. [22]) to each estimate *r*_*i*_, i.e. calculate $z_{i}:=\frac{1}{2}\text{ln}(\frac{1+r_{i}}{1-r_{i}})$. It follows from theory that approximately $z_{i}∼N(\frac{1}{2}\text{ln}(\frac{1+\rho }{1-\rho }),\frac{1}{n_{i}-3})$.
3. The estimator for the correlation is then defined as $\hat{\rho }=\text{tanh}(\frac{1}{\left|I\right|}\sum_{i=1}^{\left|I\right|}z_{i})$, using the equal correlation assumption.

$FWER_{o}^{\text{PI}}$ control is no longer guaranteed. To investigate possible inflation, the worst case $FWER_{o}^{\text{PI}}$ of the overall procedure was simulated for the cases that two or three treatments are compared with a common control, setting the per-group sample size as *n* = 5, 10, 22, 50 or 100. The testing scenario is as described in Section 4.1 and 5 ⋅ 10^5^ simulations were run for each value of *ρ* to estimate the maximum $FWER_{o}^{\text{PI}}$.

**Fig 2:**
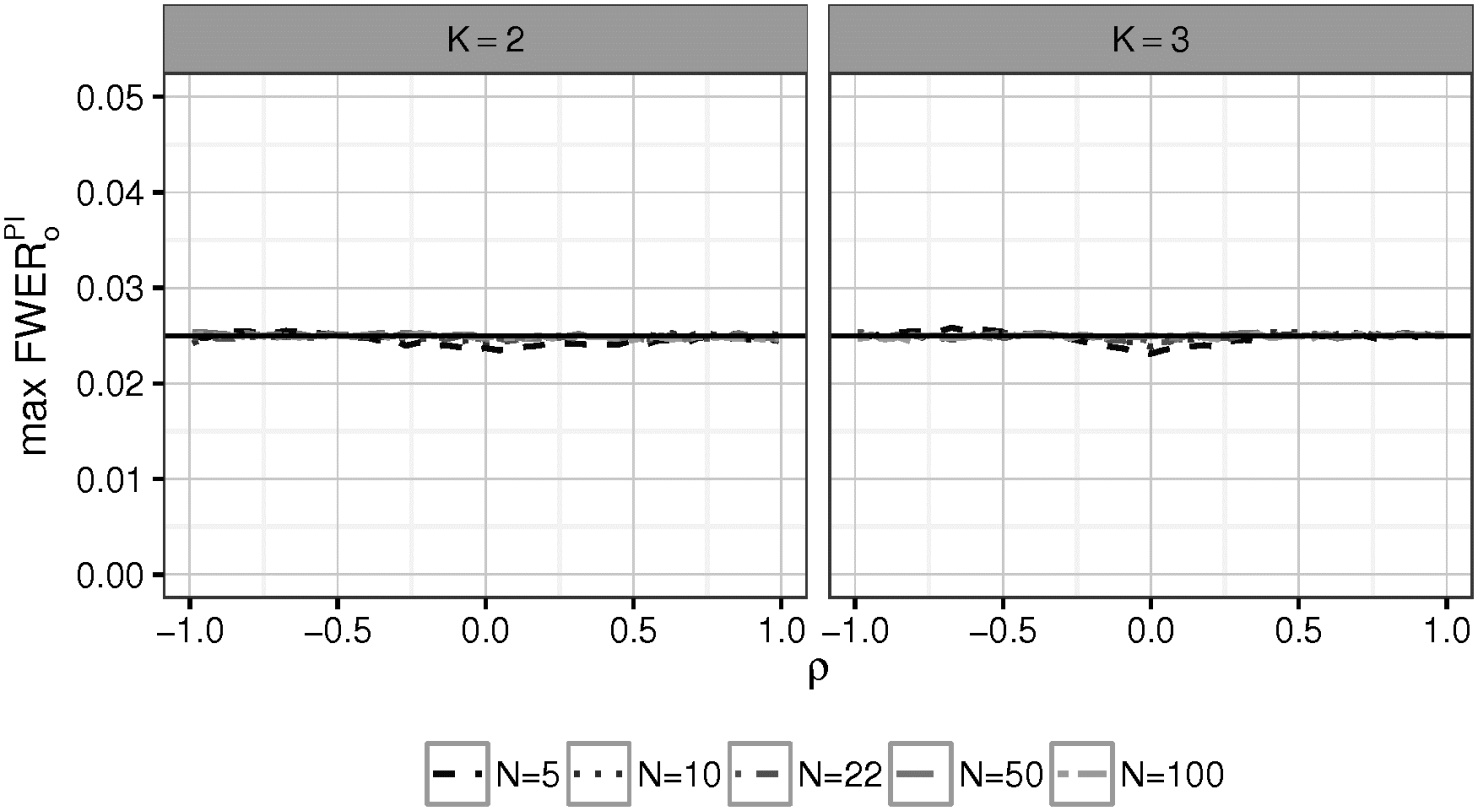
Simulated values of worst case $FWER_{o}^{\text{PI}}$ as a function of *ρ*. Two treatments were compared to one common control with equal per-group sample sizes *n*. A conservative behavior is visible around 0, when the per-group sample size is low.

In Fig 2 an incremental inflation of the maximum $FWER_{o}^{\text{PI}}$ is visible for correlations around −0.7. On the other hand, for low sample sizes the procedure is slightly conservative for *ρ* close to 0. This is reasonable since overestimating *ρ* when in truth *ρ* = 0 does no “harm” in terms of *FWER*_*o*_, because *α*_*a*_ (interpreted as a function of $\hat{\rho }$) remains then constant. Only the error of underestimation takes effect by shifting the maximum *FWER*_*o*_ slightly down. For increasing values of *n*, both effects diminish because the correlation estimate tends to be closer to the true value of *ρ*, so the impact of the conservatism due to underestimation of *ρ* is reduced. Tables of the simulated values in Fig 2 are presented in S1 Tables.

## 6 Simulation study

We perform simulations of the comparison of 3 treatments with a single control group using the procedures discussed in the previous sections to adjust for multiple comparisons, namely the natural correction (NA); the conservative correction (CO); the adjusted method for known correlation (KC); and the adjusted method for unknown correlation with the plug-in correlation estimate (PI). The underlying statistical model as well as the formulation of the null hypotheses remains as described in Section 3. The group means of the efficacy data are said to have a linear relationship if they take the following form: $\mu_{1}=\frac{1}{3}\mu_{3}$, $\mu_{2}=\frac{2}{3}\mu_{3}$. On the other hand a constant relationship is defined as: *μ*_1_ = *μ*_2_ = *μ*_3_. For the control group, the mean is always set to *μ*_0_ = 0. The true toxicity means are either constant (*θ*_1_ = *θ*_2_ = *θ*_3_ = 0), linearly increasing (*θ*_1_ = 0, *θ*_2_ = 0.5, and *θ*_3_ = 1), or linearly decreasing (*θ*_1_ = 1, *θ*_2_ = 0.5, and *θ*_3_ = 0).

For **Scenario 1**, we define constant efficacy means and linearly increasing toxicity means. In **Scenario 2**, efficacy means are linearly increasing, while toxicity means are constant. In **Scenario 3**, efficacy and toxicity means both increase linearly. **Scenario 4** describes an inverse relationship between efficacy and toxicity such that efficacy means are linearly increasing while toxicity means are linearly decreasing. In all scenarios, the variances of the efficacy and toxicity outcomes are set to 1 and the correlation between toxicity and efficacy responses is constant across treatment groups.

We evaluate the power of the overall procedure when we apply the DT with a sample size of *n* = 22 in each treatment group and on control. The measure of interest is disjunctive power, which is the probability of rejecting at least one false null hypothesis. The one-sided nominal significance level is set to *α*_nom_ = 0.025.

The simulation process is split into two parts. First, the curve of values of *α*_*a*_ corresponding to the known correlation case as plotted in the right panel of Fig 2 (dotted line for 3 vs. 1) is simulated for different correlations *ρ*. Here the number of simulation runs is 5 ⋅ 10^5^. Afterwards these simulated values are approximated by a cubic polynomial in the region of *ρ* ∈ (−1, 0). In the second step of the simulation study, patient responses are sampled from the bivariate normal model and the fraction of simulated trials leading to the rejection of at least one false null hypothesis is counted. For the KC and PI procedures we use the simulated and smoothed *α*_*a*_ curve from the first step. The number of simulation runs in the second part is 20000.

### 6.1 Power

**Fig 3:**
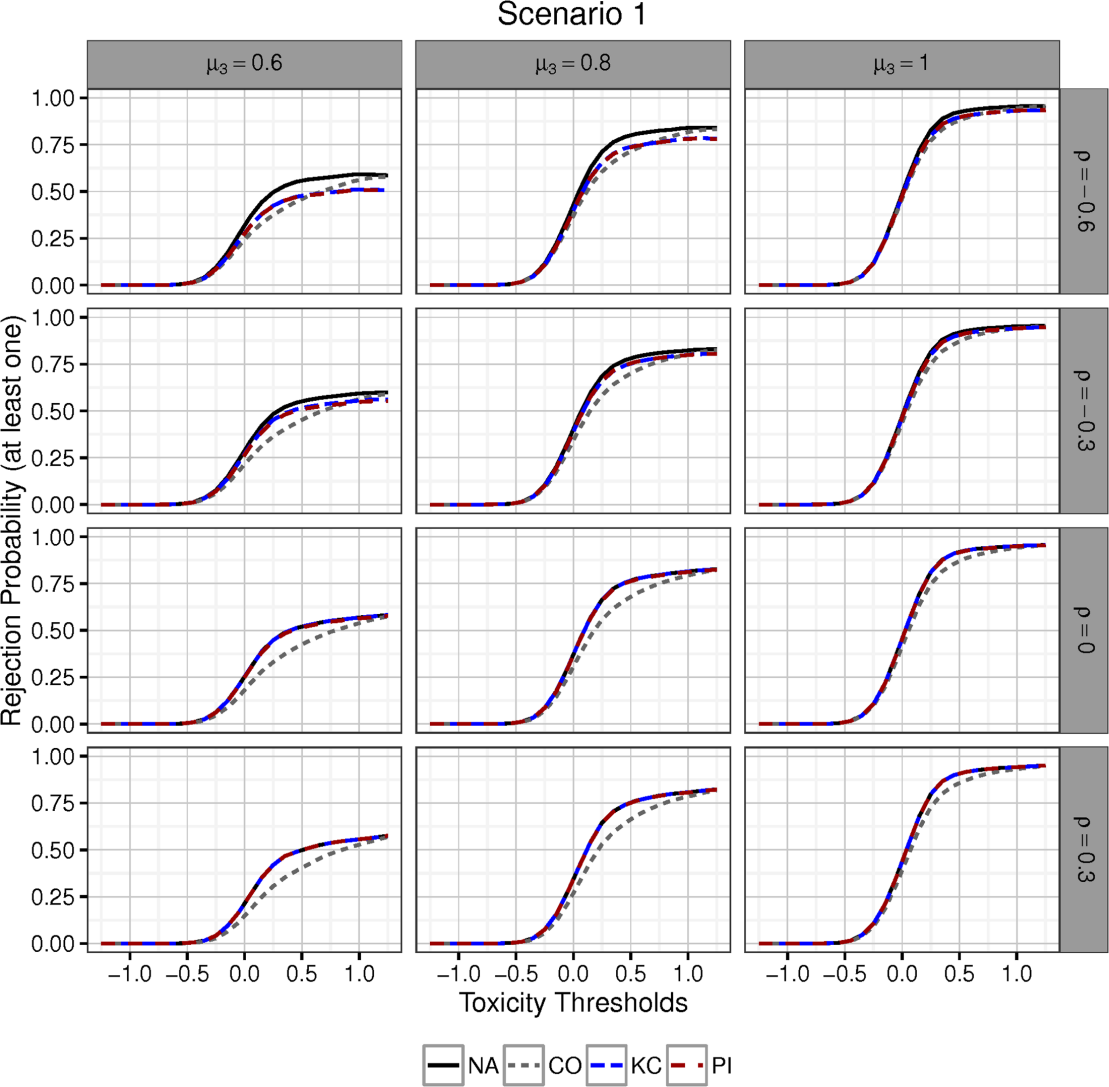
Scenario 1; Power of different two-step procedures as a function of the toxicity thresholds. High values of the toxicity threshold (defined to be the same in each group) imply more treatments are selected for efficacy testing. Rows correspond to different values of *ρ* ∈ {−0.6, −0.3, 0, 0.3}; columns correspond to values of *α*_3_ ∈ {0.6, 0.8, 1} in the linear scenario (see also the main text). As *ρ* increases, the region where the adjusted approaches dominate the conservative procedure in terms of power, increases in area. This behaviour accelerates for higher values of *α*_3_.

Fig 3 shows the results for Scenario 1. In the first column of figures, *μ*_3_ is set to 0.6, whereas in the second and third columns we have *μ*_3_ = 0.8 and *μ*_3_ = 1, respectively. Rows of figures are characterised by different values of the true correlation *ρ*, specifically −0.6, −0.3, 0, and 0.3. On the horizontal axis, the toxicity threshold (assumed to be equal across treatment groups) is drawn. On the vertical axis, we have the probability that the overall procedure rejects at least one null hypothesis.

Rejection probabilities always converge to 0 as the toxicity thresholds becomes sharper since eventually no treatment is selected and taken forwards for efficacy testing. However, rejection probabilities interpreted as a function of varying toxicity thresholds are not necessarily monotone. This can be explained by the fact that increasing the toxicity thresholds increases the expected number of treatments selected, but more treatments in the efficacy testing stage also implies sharper efficacy thresholds. Despite this, no large deviations from the rule-of-thumb that increasing the toxicity thresholds leads to increased disjunctive rejection probabilities are observed.

Regarding the curves for the two-step procedure using the natural correction and the procedure using the conservative correction, it is visible that at a certain point the former procedure starts to outperform the latter. This is the case when the safety selection rule begins to select treatments with non-negligible probability. At this point the efficacy testing boundaries for these procedures begin to differ and their power curves diverge. Increasing the toxicity thresholds further, eventually we find that all treatments will be selected with a high probability. The natural and conservative procedures then use the same boundaries in the efficacy testing step and both power curves converge towards each other again.

When we use the procedure for known correlation, following the steps set out in Section 5.1, then its power will never converge towards that of the natural procedure when *ρ* < 0. This is the case because a lower nominal significance level is assumed for the boundary calculation in the efficacy testing step (as can be seen in Fig 1, right). Meanwhile, the adjusted natural procedure outperforms the conservative procedure in terms of power in settings where all treatments are dropped with high probability. When *ρ* = −0.6, the gains in power made by the adjusted natural approach on the conservative procedure are small relative to the losses in power incurred when all treatments are regarded as safe. For increasing (negative) *ρ*, the power curve of the KC approach converges to that of the natural procedure, making the region of superiority over procedure CO broader. For non-negative correlation, KC is identical to NA as explained in Section 5, and therefore these procedures have the same power. Regarding the power of procedure PI, its power curve is very close to that of procedure KC.

**Fig 4:**
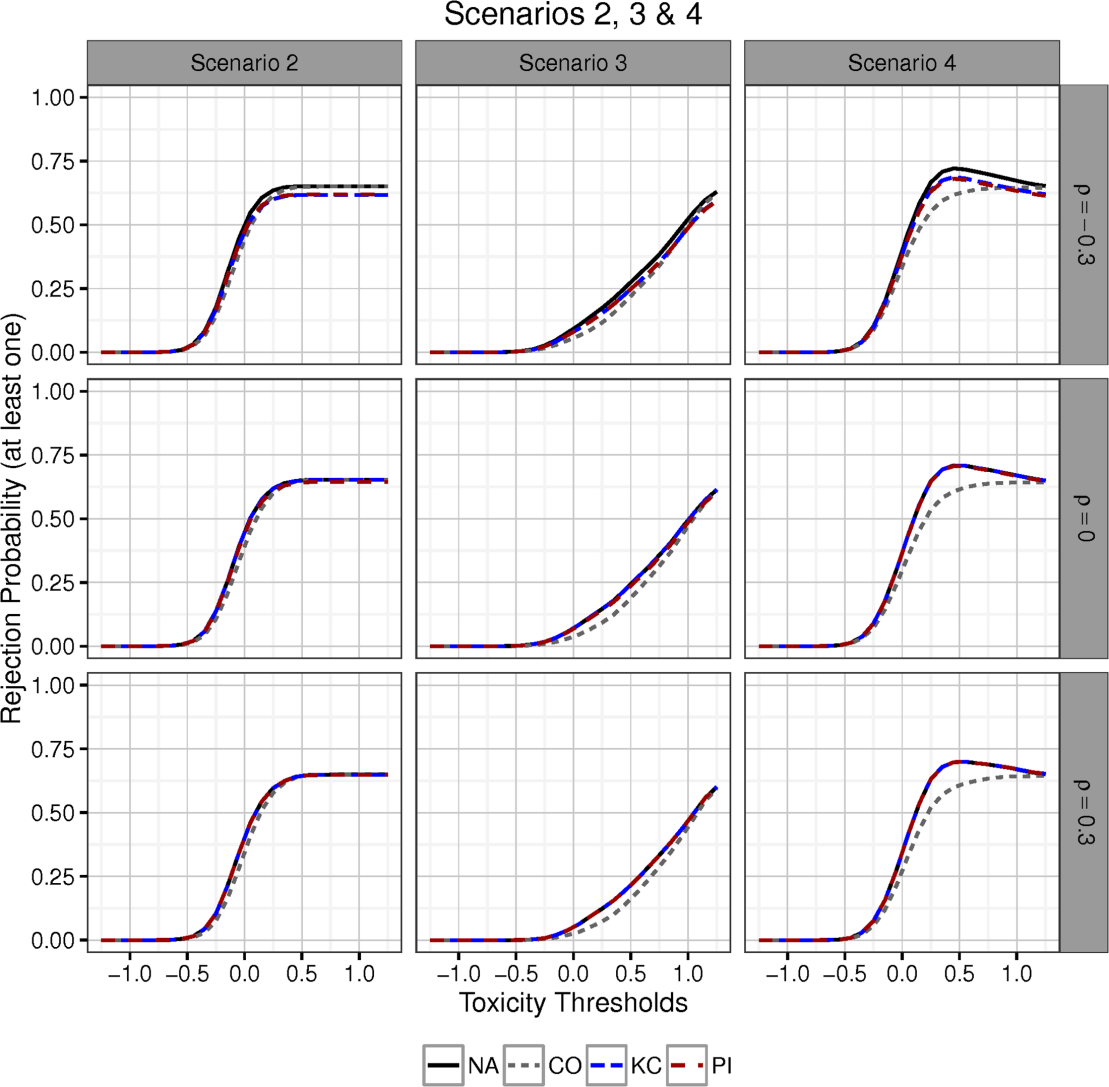
Power in Scenario 2 (left column), Scenario 3 (middle column) and Scenario 4 (right column). Different efficacy/toxicity patterns lead to varying gains in power over procedure CO. Rows of figures are generated setting *ρ* = −0.3, 0 or 0.3. In all scenarios, *μ*_3_ = 0.8. The smallest gains in power are made in Scenario 2, which represents an all-or-none selection situation. Deviating from this setting leads to gains in power in the area where dropping treatments is expected (Scenarios 3 and 4). The largest power gains are seen in Scenario 4 where the slopes of the linear configurations of the toxicity and efficacy means are of different signs.

For scenarios 2, 3 and 4 we set *μ*_3_ = 0.8 and *ρ* ∈ {−0.3, 0, 0.3}. In Scenario 2 of Fig 4 (left column), toxicity means are equal but efficacy means are linearly increasing. As the toxicity threshold increases, the probability that all three treatments are selected rapidly increases from 0 to 1. This is the case because toxicity means are all equal in this scenario and likely either all the treatments satisfy the selection criterion or none do. Differences in toxicity sample means are then due to random variation alone. Therefore in this scenario an all-or-none treatment selection situation is likely. As we have noted for Scenario 1, the other testing approaches dominate procedure CO in situations where dropping *some* (but not all) treatments is likely. Therefore the size of the region where procedures KC and PI dominate CO is small and the margin of dominance is negligible.

In Scenario 3 (Fig 4, middle column), toxicity means are linearly increasing. As the toxicity threshold is gradually relaxed, one-by-one the three treatments can be deemed safe in the sense that they will satisfy the safety selection criterion with high probability. Therefore, since the expected number of selected treatments increases incrementally with the safety threshold, the slope of the power curve is less steep compared to Scenario 2. On the other hand gains in power of NA, KC, and PI over procedure CO are larger than those seen in Scenario 2.

In Scenario 4, (Fig 4, right column), efficacy means are increasing across treatment groups 1, 2 and 3, while toxicity means are decreasing. This mimics the situation where toxicity might negatively impact on efficacy. In this scenario treatments with higher efficacy are more likely to be selected. Similar to scenario 3, since toxicity means for treatments are not all equal, we see a more gradual increase in power compared with Scenario. For intermediate values of the safety threshold, the treatments most likely to be selected are those with higher efficacy. Therefore, relaxing the boundaries of the DT results in a greater increase of power compared with other testing scenarios (where safer treatments are also less efficacious) and differences between the power curves of procedure CO and the other procedures are more marked.

In all scenarios, as the toxicity threshold is relaxed the expected number of null hypotheses that are tested increases. What can be gained by applying the proposed two-step procedures in comparison to the conservative procedure in terms of disjunctive power, depends on the exact configuration of toxicity and efficacy means. It can be seen from Figs 3 and 4 that changes in the safety selection rule have a similar impact on the operating characteristics of all MTPs, although clearly some procedures are more powerfull than others due to differences in the exact choice of efficacy boundaries applied to the selected null hypotheses. For example the possibilities for power gains compared to the conservative procedure increase, if treatments are selected, that also have the largest treatment effect. This situation is reflected in Scenario 4.

### 6.2 Impact of varying the sample size

**Fig 5:**
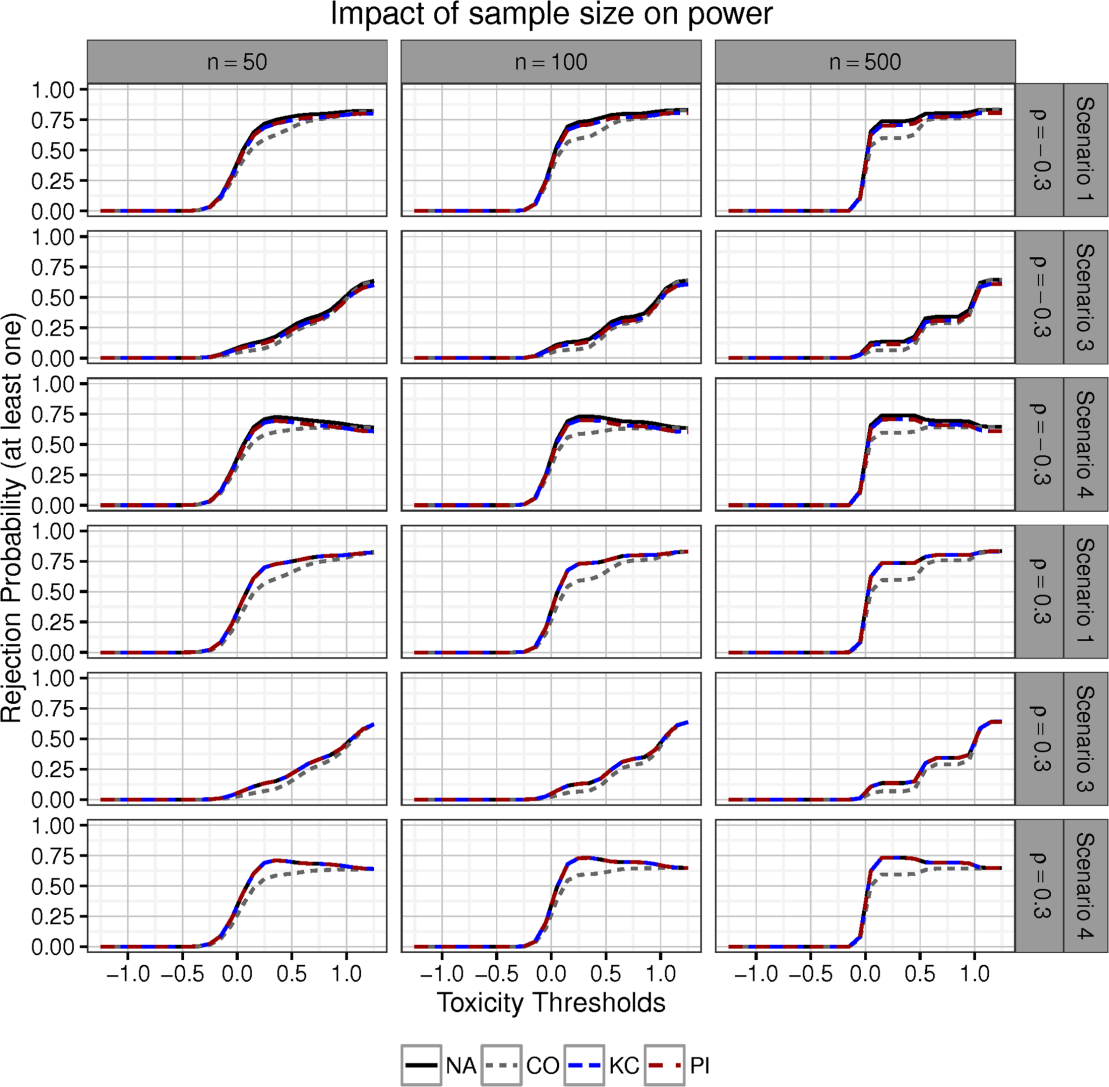
Power of the four two-step procedures under varying toxicity thresholds and (per-group) sample sizes in Scenarios 1, 3, and 4. For each group size, the effect size *μ*_3_ is chosen as the difference a single treatment comparison can detect with power 0.9. The correlation is set as *ρ* ∈ {−0.3, 0.3}.

Fig 5 plots the power of the two-step procedures under varying toxicity thresholds. Rows consider Scenario 1, Scenario 3 and Scenario 4, and different values of the correlation coefficient (*ρ* ∈ {−0.3, 0.3}). In the left column, the per-group sample size is *n* = 50 and *μ*_3_ is set equal to 0.52. In the middle (*n* = 100) and right (*n* = 500) column, we set *μ*_3_ = 0.368 and *μ*_3_ = 0.164, respectively. Values of *μ*_3_ are chosen such that a single treatment-control comparison has power 0.9 of detecting the effect *μ*_3_ for the given group size. As *n* increases, the power curves of the two-step procedures essentially converge towards step functions. The reason for this is that with increasing sample size, the true toxicity means can be estimated with greater accuracy. As the sampling error of the group sample mean toxicities decreases, we can clearly distinguish between ‘safe’ and ‘toxic’ treatments; thus, varying the toxicity threshold on an interval between two true toxicity means has little impact on the treatment selection procedure in terms of the expected number selected and the identity of the treatments selected.

From Figs 3–5 it can be seen, that the prespecified sample sizes *n* ∈ {22, 50, 100, 500} per group are large enough to achieve a high correspondence between the KC and the PI procedure. One intuitive explanation for this reason is that over- und underestimation of *ρ* which occurs by using the empirical correlation coefficient, roughly balance out, as explained in Section 5.2. On the other hand the conservativeness of the PI procedure for small sample sizes around *ρ* = 0 as seen in Fig 2, suggests a small power loss compared to PI in this setting. In S1 Fig a power curve is plotted for *ρ* = 0 and *n* = 5 in the situation of Scenario 1 with *μ*_3_ again chosen such, that the power for a single treatment-control comparison is 0.9. The graph shows a small power loss of the PI procedure due to estimation error.

### 6.3 Impact of the selection probability

**Fig 6:**
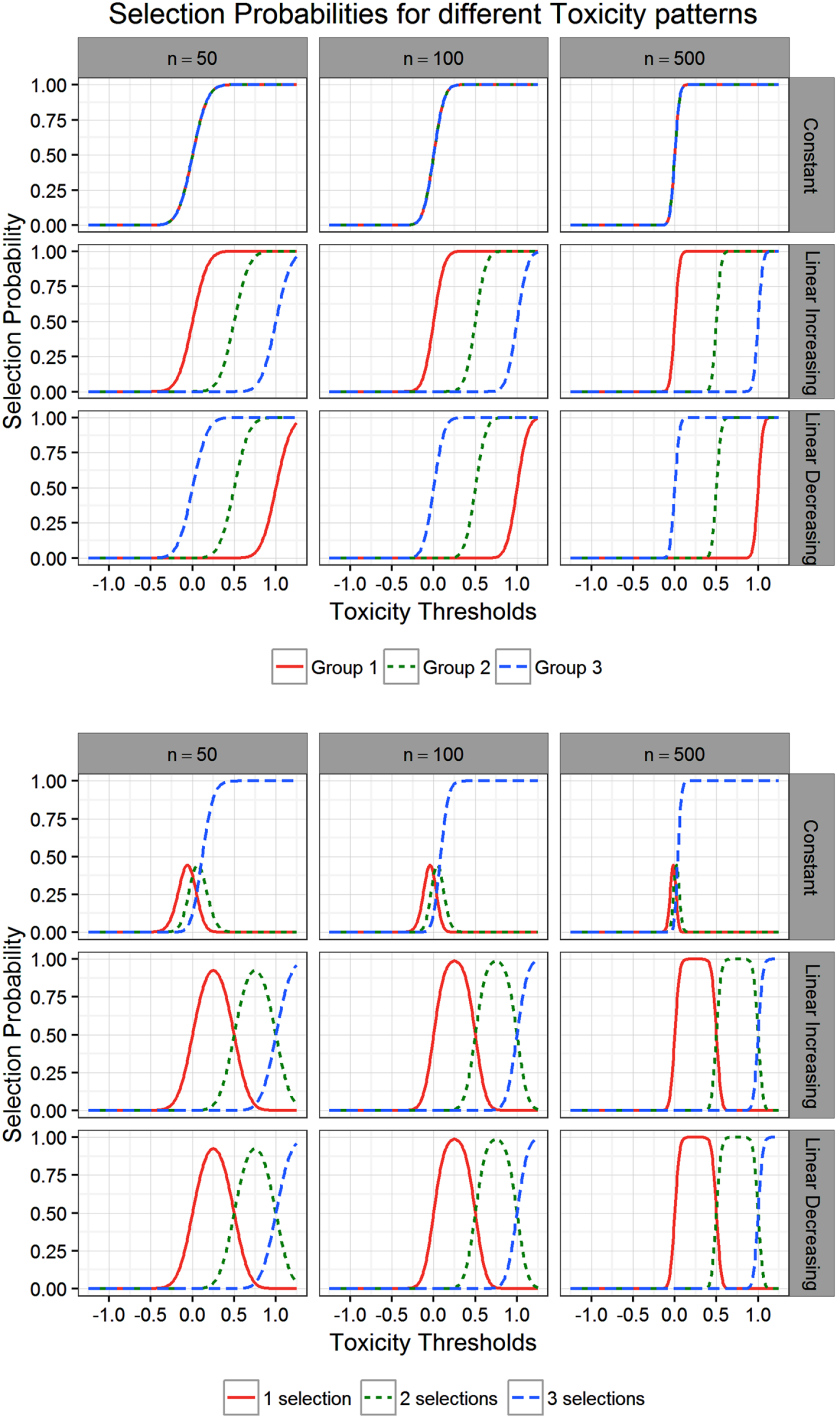
Selection probabilities for different toxicity patterns. In the upper plot the probabilities of selecting treatments 1, 2 or 3 are drawn. The probabilities of selecting exactly 1, 2, or 3 treatments are drawn in the lower plot.

The behaviour of the power curves seen in Fig 5 can be explained by taking a closer look at the safety selection procedure. The top plot of Fig 6 shows the probabilities of selecting different treatment groups. Rows correspond to constant (Scenario 2), linear increasing (Scenario 1 and 3), and linear decreasing (Scenario 4) toxicity means. The columns are characterised by different sample sizes. For increasing sample sizes, estimation of the group toxicity means becomes more precise, which leads to a decreased variability in the selection process and therefore to steeper power curves. At the boundaries 0, 0.5, and 1, the probabilities of being selected are sharply increasing for the corresponding group. In the lower plot of Fig 6, the probabilities of selecting at exactly 1, 2, or 3 treatments are drawn. For larger sample sizes, the area of overlap between these curves diminishes. This is the reason for the plateaus visible in Fig 5.

## 7 Discussion

We have considered two-step procedures, where safety selection precedes efficacy testing of multiple hypotheses using naive Dunnett boundaries and adjusting just for the number of selected treatments. For such a procedure we demonstrated that the *FWER* of the overall procedure may exceed the nominal level *α*. The size of this deviation depends on the unknown distribution of the toxicity measurements and pre-defined toxicity thresholds. In addition, the dependency structure of the efficacy and toxicity outcomes determines the size of the maximum possible *FWER*_*o*_. We assumed a bivariate normal model for efficacy and toxicity measurements, in which case the dependency structure is captured by correlations. Here toxicity was represented by a normally distributed variable. In practice, the adverse effects of a drug on the kidneys, liver or heart are often monitored by continuous parameters such as laboratory values or variables from an ECG, respectively.

A major finding of the present work is that selection of treatments based on observed safety data is indeed possible and multiple comparison procedures such as Dunnett or Bonferroni tests which adjust only for the number of selected treatments will control the *FWER*_*o*_, so long as toxicity and efficacy measurements are (positively) associated in the sense of [21]. Intuitively this can be explained by the fact that in case of a positive association, treatments that show high efficacy are more likely to be dropped. Or, conversely, if a treatment is selected on the basis of lower toxicity, under the null hypothesis it will be more likely that lower efficacy is observed too. In the bivariate normal model, this is the case when the correlation between toxicity and efficacy outcomes are non-negative in all treatment groups. Clearly, if all correlations are zero, selection is independent of efficacy testing. Then a Dunnett adjustment for the remaining treatments controls the FWER and depending on the safety selection rule, it may become conservative.

In the case that the correlation between efficacy and toxicity is negative for at least one treatment group, dropping such a treatment group would imply dropping a treatment which likely has poor efficacy measurements relative to those on the other treatments. If then for the remaining treatments the efficacy testing thresholds are relaxed, the overall *FWER* will be inflated. In our particular setting, we have shown that this inflation will be maximized when all correlations between efficacy and toxicity approach the value −1. We proposed an adjusted approach, in which we change the nominal *α*-level to be used in the Dunnett adjustment for efficacy testing depending on the correlation. This means that if one assumes no or positive correlations, then the resulting two-step procedure is exactly as described above. It is sufficient to take Dunnett boundaries adjusting for the number of selected treatments at nominal level *α*. Assuming negative correlations will result in Dunnett tests adjusting for the number of selected treatments at a lower nominal *α*-level. When the correlation is unknown, which will often be the case in clinical research, a reasonable lower boundary for the correlation may be assumed. For example in oncology it is occasionally assumed that the correlation between efficacy and toxicity is non-negative. On a trial level, this is shown by [23]. A reasonable lower boundary for the correlation is then 0. According to our results it is then possible to drop treatments due to toxicity and calculate the Dunnett boundaries at nominal level *α*_*nom*_ adjusting for the number of selected treatments only without inflating the *FWER*.

Alternatively it is suggested to estimate the correlation and use this estimate to adjust the nominal *α*-level. This is especially useful, if correlations are assumed to be equal, which leads to a pooling of the different groups in terms of correlation estimation. Simulations have shown, that such a strategy is adequate even if results on exact overall *FWER* control or inflation are missing. Compared to the conservative approach (always adjusting for all a-priori defined comparisons) the proposed two-step testing strategies result in higher power for non-negative correlations. For negative correlations there is a gain in power if it is very likely that treatments have to be dropped due to toxicity. However, if all treatments are sufficiently safe (and likelihood of dropping is negligible), the proposed two step-testing procedures will have a loss of power as a lower nominal level alpha to be used in the Dunnett test. This is the price to be paid for adjusting just for the number of selected treatments. However, negative correlations may be implausible in many practical situations.

In the planning phase of a clinical trial, the first consideration is regarding the correlation between toxicity and efficacy. In accordance to the arguments presented before, if the assumption on positive or zero correlation is justified, then the use of the natural procedure is reasonable and simulation studies for different scenarios as performed in Section 6 support decisions regarding the planned per-group-sample sizes. Justification for non-negative correlation could be derived from a scientific understanding of the relationship between two endpoints, or on data from previous trials which measured the two endpoints. When an assumption on positive correlation is not plausible, considerations on reasonable values of the correlation may enable the use of the correlation-adjusted procedure. Another possibility is to estimate the correlation. In each of these cases, a comprehensive simulation study would typically be performed ahead of time to quantify the operating characteristics of the proposed testing procedure in scenarios consistent with any assumptions about the correlation coefficient, and to establish the robustness of properties to deviations from these assumptions. As a sidenote it should be mentioned, that at the end of the study prefixed and biologically motivated toxicity thresholds are used to perform the statistical analysis. Simulations for varying toxicity thresholds as performed in Section 6 are essential to determine the sample sizes required and to evaluate the impact of the proposed selection rule (and the underlying toxicity thresholds) on the operating characteristics for different efficacy and toxicity means.

In summary the proposed two-step procedures are a way to integrate both safety and efficacy aspects in a more formal way for decision making on the benefit and risk ratio of new treatments. All safety and efficacy evaluations are performed at the end of the study, when for all patients all safety and efficacy data are available. Treatments are first screened on the basis of safety, and only those deemed to be sufficiently safe are considered for the efficacy testing step. The arguments provided in this paper can be extended allowing to perform the safety selection step already earlier and more frequently in the trial, e.g., for clinical trials where a data and safety monitoring board will perform periodic safety monitoring when the trial is still on-going and more patients still have to be included. For example in more “traditional” adaptive seamless designs [24–26] treatment selection is suggested to be conducted at an adaptive interim analysis, where only a first cohort of patients have been included in the trial. If treatments are selected, a further cohort with new patients have to included and randomized to the selected treatment arm. This is in contrast to the proposed two-step procedure in this paper, where safety selection is conducted at the end. This prohibits a direct head-to-head comparison as selection rules would have to differ substantially using different amount of data. Therefore part of our future work is to extend our two-step procedures to allow interim safety selection as well as a comparison to adaptive seamless designs [24, 25] using the same amount of data for selection. Further investigations may focus on other types of safety data such as, binary or ordinally scaled, where results of positive association may be applicable as well, for example if the probability of having an adverse event increases with efficacy. A key point of the two-step procedure is that the toxicity boundary have to be fixed in advance and no efficacy data are involved when deciding which treatments shall be dropped.

## Appendix

### Proof of Theorem 1

We will prove Theorem 1 in Section 4.1. In this proof we will apply the Slepian theorem (see, for example, the Appendix of [27]) which states that for every *k*-dimensional random vector **z** following a multivariate normal distribution with zero mean and unit variance in each component and correlation matrix **R**, the probability *P*(*z*_1_ ≤ *c*_1_, …, *z*_*k*_ ≤ *c*_*k*_) is a strictly increasing function of every off-diagonal entry of **R**.

*Proof*. According to Eq (1), the *FWER*_*o*_ is a sum with addends of the following form:

$$\begin{array}{c} P(\underset{i\in S}{⋂}\left\{{\bar{y}}_{i}\leq b_{i}^{y}\right\}\cap \underset{j\in I\setminus S}{⋂}\left\{{\bar{y}}_{j}>b_{j}^{y}\right\})-C\cdot P(\underset{j\in I\setminus S}{⋂}\left\{{\bar{y}}_{j}>b_{j}^{y}\right\}) \end{array}$$ (2)

where $C:=P({⋂}_{i\in S\cap J}\left\{{\bar{x}}_{i}-{\bar{x}}_{0}<b_{i,S}^{x}\right\}\cap {⋂}_{i\in S}\left\{{\bar{y}}_{i}\leq b_{i}^{y}\right\})$ (here we made use of the independence between measurements in different groups). Following Slepian’s result, -C is monotonically decreasing in *ρ* and the statement is proofed for the ZT framework. For the DT, all probabilities in Eq (2) are conditional to an independent variance estimate (and also the thresholds $b_{i,S}^{x}$ now depend on these estimates). On all these probabilities the monotonicity statement remains to be true. Averaging over all possible values then shows, that our result is true in the DT framework as well.

### Proof of Theorem 2

In what follows we will provide and prove a sufficient condition for *FWER*_*o*_ control of a procedure that controls the FWER in the efficacy testing step. To do this we first need the notion of association as defined in [21]:

*By definition the random variables* (*Q*_1_, …, *Q*_*N*_) =: **Q** *are associated if and only if*

$$\begin{array}{c} E\left[f\left(Q\right)\cdot g\left(Q\right)\right]\geq E\left[f\left(Q\right)\right]\cdot E\left[g\left(Q\right)\right] \end{array}$$ (3)

*for every componentwise non-decreasing functions f and g*.

*Remark* 1. From the definition it follows immediately, that non-decreasing transformations of associated random variables are also associated. Another intuitively clear property of association is, that the set of a single variable is associated. Furthermore, if two sets of associated random variables are independent, then the union is also associated. All of these properties are proven in [21].

In preparation of proving Theorem 2, two lemmata are presented:

**Lemma 1**. *Let* ∅ ≠ *J* ⊆ *I* *be the index set of inefficacious treatments. The maximum FWER_o_ is controlled at level α_nom_ for a given multiple comparison procedure, if FWER(S)* ≤ *α_nom_ (that means* $E[\varphi_{J}^{S}]\leq \alpha_{nom}$) *and* $E[\varsigma_{S}\cdot \varphi_{J}^{S}]\leq E[\varsigma_{S}]\cdot E[\varphi_{J}^{S}]$ *both hold for* $\forall S\in M_{J}:=\left\{S\subseteq I:S\cap J\neq \emptyset \right\}$.

*Proof*. We first note that only one of the binary indicators *ς*_S_ with $S\in M_{J}$ can have the value 1 at once. Here $M_{J}$ is the set of all possible selections, that contain at least one true null-hypothesis. The sets $S\notin M_{J}$ are not relevant for type I error control. We have seen earlier in Eq (1) that the *FWER* of the overall procedure can be written as $FWER_{o}=\sum_{S\in M_{J}}E[\varsigma_{S}\cdot \varphi_{J}^{S}]$. Because $E[\varsigma_{S}\cdot \varphi_{J}^{S}]\leq E[\varsigma_{S}]\cdot E[\varphi_{J}^{S}]$ holds for each addend it holds $\sum_{S\in M_{J}}E[\varsigma_{S}\cdot \varphi_{J}^{S}]\leq \sum_{S\in M_{J}}E[\varsigma_{S}]\cdot E[\varphi_{J}^{S}].$ The factor $E[\varphi_{J}^{S}]$ is bounded from above by *α*_nom_. Therefore it holds

$$\begin{array}{ll} \sum_{S\in M_{J}}E\left[\varsigma_{S}\cdot \varphi_{J}^{S}\right] & \leq \alpha_{\text{nom}}\cdot \left(\sum_{S\in M_{J}}E\left[\varsigma_{S}\right]\right)\\ & \leq \alpha_{\text{nom}}. \end{array}$$

The last inequality follows from the fact that $\sum_{S\subseteq M_{J}}E[\varsigma_{S}]$ is just a sum of probabilities of disjoint events and therefore less or equal 1 (in general equality only holds, if *J* = *I*).

**Lemma 2**. *Let* ∅ ≠ *J* ⊆ *I be the index set of inefficacious treatments. For all subsets S* ⊆ *I let the families of indicators* (*τ_i_*)*_i_*_∈_*_I_* and ${\left(\eta_{i}^{S}\right)}_{i\in I}$ *have the following properties*:

1. *The vector* (*τ*_*i*_)_*i*∈*I*∖*S*_ *is independent to the vector* $\left({\left(\tau_{i}\right)}_{i\in S},{\left(\eta_{i}^{S}\right)}_{i\in S}\right)$.
2. *The set* $\left\{\tau_{i}:\;i\in I\right\}\cup \left\{\eta_{i}^{S}:i\in I\right\}$ *is associated*.

*Then* $\forall S\in M_{J}$ *it holds*

$$\begin{array}{c} E\left[\varsigma_{S}\cdot \varphi_{J}^{S}\right]\leq E\left[\varsigma_{S}\right]\cdot E\left[\varphi_{J}^{S}\right]. \end{array}$$ (4)

*Proof*. Let $S\in M_{J}$ be fixed. It holds $E[\varsigma_{S}\cdot \varphi_{J}^{S}]=E[\prod_{j\in I\setminus S}\tau_{j}]\cdot E[(\prod_{i\in S}(1-\tau_{i}))\cdot \varphi_{J}^{S}]$ due to the definition of *ς*_S_ and property 1. We focus on the right-hand factor $E[(\prod_{i\in S}(1-\tau_{i}))\cdot \varphi_{J}^{S}]$ and note that *f* := (−1) ⋅ ∏_*i* ∈ *S*_(1 − *τ*_*i*_) and $g:=\varphi_{J}^{S}$ both interpreted as functions of the *τ*_*i*_ and $\eta_{i}^{S}$ are monotonically non-decreasing. Due to the association assumption Eq (3) it therefore holds $E[(\prod_{i\in S}(1-\tau_{i}))\cdot \varphi_{J}^{S}]\leq E[\prod_{i\in S}(1-\tau_{i})]\cdot E[\varphi_{J}^{S}]$ (note that the minus sign in *f* reverses the inequality sign). By again using the independence property 1, Eq (2) is proven.

We now have the tools to prove **Theorem 2**:

*Proof*. At first, we consider the ZT framework. We are going to prove that under the stated assumptions Lemma 2 holds. Then Lemma 1 can be applied. We set $\tau_{i}:=1_{\left(b_{i}^{y},\infty \right)}\left({\bar{y}}_{i}\right)$ and $\eta_{i}^{S}:=1_{\left(b_{i,S}^{x},\infty \right)}\left({\bar{x}}_{i}-{\bar{x}}_{0}\right),\forall i\in I$ and for fixed *S* ⊆ *I*. The boundaries $b_{i,S}^{x}$ are chosen such, that the FWER is controlled at level *α*_nom_ in the efficacy testing step for every *S* ⊆ *I*. We know that by construction the vectors $\left({\bar{y}}_{i}:i\in I\setminus S\right)$ and $\left({\bar{x}}_{i}-{\bar{x}}_{0},{\bar{y}}_{i}:i\in S\right)$ are independent. This also holds for the corresponding vectors of the indicators *τ*_*i*_ and $\eta_{i}^{S}$, which verifies property 1 in Lemma 2.

It is easy to see that $\text{Cov}({\bar{y}}_{i},{\bar{x}}_{i}-{\bar{x}}_{0})=\frac{\rho_{i}\sigma \omega }{n_{i}}\geq 0$ and for *i* ≠ *j* it holds that $\text{Cov}({\bar{x}}_{i}-{\bar{x}}_{0},{\bar{x}}_{j}-{\bar{x}}_{0})=\frac{\sigma^{2}}{n_{0}}\geq 0$, and therefore all pairwise correlations are non-negative. From [28] we know that positively correlated multivariate normal random variables are associated. This proves the association of the set of random variables $\left\{{\bar{y}}_{i}:\;i\in I\right\}\cup \left\{{\bar{x}}_{i}-{\bar{x}}_{0}:\;i\in I\right\}$. According to Remark 1, the same is then true for above defined indicators, which are monotonically non-decreasing functions. This concludes the proof of Lemma 2. After applying Lemma 1, this proofs *FWER*_*o*_ control at level *α*_nom_ in the *ZT* scenario, if all the correlations *ρ*_*i*_ are non-negative.

For the proof of the DT framework by *V*_*i*_ we denote the unbiased variance estimator in group *i*, that is $V_{i}:=\frac{1}{n_{i-1}}\overset{n_{i}}{\underset{i=1}{\sum }}{\left(x_{ij}-{\bar{x}}_{i}\right)}^{2}$. The test statistics of the DT use the overall variance estimator, which is function of the group variance estimates $V^{S}:=\sum_{I\cup \{0\}}\frac{n_{i}-1}{\sum_{I\cup \{0\}}(n_{i}-1)}V_{i}$. The multivariate extension of a fundamental result in statistics states, that the sample covariance matrix (consisting of *V*_*i*_) defined for the data matrix of both efficacy and toxicity is independent of the corresponding sample mean vector (see for example [22] p. 48). The same is then true for overall variance *W*^*S*^ := −*V*^*S*^, which is just a function of the sample covariance matrix, and thererfore ${\left({\bar{y}}_{i}\right)}_{i\in I\setminus S}$ and $\left({\left({\bar{x}}_{i}-{\bar{x}}_{0}\right)}_{i\in S},{\left({\bar{y}}_{i}\right)}_{i\in S},W^{S}\right)$ are independent. *W*^*S*^ just influences the thresholds $b_{i,S}^{x}$ in $\eta_{i}^{S}$ and therefore by using the same arguments as in the ZT case, the fulfilment of property 1 in Lemma 2 is shown.

For property 2 we have to note that *W*^*S*^ itself forms an associated set and due of independence the same is then true for the set $\left\{{\bar{y}}_{i}:\;i\in I\right\}\cup \left\{{\bar{x}}_{i}-{\bar{x}}_{0}:\;i\in I\right\}\cup \left\{W^{S}\right\}$. For increasing *W*^*S*^ (which means decreasing *V*^*S*^), $b_{i,S}^{x}$ is decreasing and therefore $\eta_{i}^{S}$ is non-decreasing. For the remaining the argument from the ZT case apply. This proves property 2 in Lemma 2.

*Remark* 2. It can be seen from the proof, that in the DT setting the assumption of equal group variances as stated in Section 3.2 is not used, except that it implies the use of the group variance estimator *V*^*S*^. This represents the classical situation of the Dunnett test. For the straightforward generalization of different variances, Theorem 2 remains true, if the variances are estimated by the *V*_*i*_ (or functions of these). For the ZT situation, *σ* can be substituted by *σ*_*i*_ in the proof.

### Extensions

The bivariate normal model also serves as a basis for an extension of the ZT approach for binary toxicity indicators, e.g. adverse events. This can be done by defining indicators for patient *j* in group *i*: $z_{ij}:=1_{\left\{(b_{ij}^{z},\infty )\right\}}(y_{ij})$ with patient specific thresholds $b_{ij}^{z}$, where the normally distributed *y*_*ij*_ are now interpreted as latent variables. Safety selection would then be based on the proportion of adverse events. Because the application of the indicator function on the latent variables *y*_*ij*_ preserves association and independence, Theorem 2 can be easily extended for the present case.

Extending the DT setting to binary toxicity data is more complicated since the variance estimator and the proportion of adverse events are not independent any more. In this case the fulfilment of property 2 of Lemma 2 cannot be shown by considering the union of the independent associated sets.

Another possible extension for safety selection to consider the toxicity measurements of a treatment group relative to the control measurements, in contrast to examine safety based on the groups toxicity data only. If the control efficacy data is correlated with the group efficacy data, the arguments in Theorem 2 based on independence again cannot be applied.

For the latter two cases, for the possibility of *FWER*_*o*_ control formal proofs cannot be derived. These scenarios remain an open topic for future research.

## Supporting information

S1 Tables Simulated maximum FWERs, that are presented in Fig 2. (PDF)

S1 Fig Power for *ρ* = 0 and *n* = 5. (PDF)

S1 Example Hypothetical example of the two-step procedure. (PDF)
