# Supplementary material for: Many-to-one comparisons after safety selection in multi-arm clinical trials
Source: PLoS One. 2017 Jun 26;12(6):e0180131. doi: 10.1371/journal.pone.0180131 (PMC5484516; doi:10.1371/journal.pone.0180131)
Supplement: S1 Tables — (PDF) [file pone.0180131.s001.pdf]

S1 Tables: Supporting Information for ‘Many-to-One Comparisons after Safety Selection in Multi-Arm Clinical Trials’

Simulated maximum FWERs, that are presented in Fig 2

The following tables provide simulated maximum FWERs, that are plotted in Figure 2 in the publication (Subsection 5.2) for $K = 2$ (Table 1) and $K = 3$ (Table 2) experimental groups.

**Max. FWER (K=2)**					
	n				
ρ	5	10	22	50	100
-0.99	0.0249	0.0242	0.0247	0.0253	0.0251
-0.90	0.0252	0.0250	0.0246	0.0252	0.0250
-0.81	0.0255	0.0251	0.0249	0.0253	0.0248
-0.72	0.0253	0.0252	0.0249	0.0251	0.0247
-0.63	0.0254	0.0250	0.0250	0.0252	0.0248
-0.54	0.0252	0.0250	0.0253	0.0251	0.0249
-0.45	0.0248	0.0248	0.0251	0.0248	0.0250
-0.36	0.0245	0.0249	0.0250	0.0253	0.0251
-0.27	0.0240	0.0247	0.0252	0.0253	0.0250
-0.18	0.0238	0.0246	0.0251	0.0250	0.0252
-0.09	0.0238	0.0244	0.0249	0.0251	0.0251
0.00	0.0237	0.0243	0.0246	0.0246	0.0249
0.09	0.0236	0.0245	0.0245	0.0249	0.0248
0.18	0.0240	0.0248	0.0249	0.0247	0.0250
0.27	0.0242	0.0250	0.0249	0.0249	0.0249
0.36	0.0240	0.0248	0.0249	0.0248	0.0248
0.45	0.0241	0.0249	0.0249	0.0247	0.0246
0.54	0.0241	0.0252	0.0244	0.0246	0.0248
0.63	0.0245	0.0253	0.0250	0.0249	0.0246
0.72	0.0248	0.0253	0.0246	0.0248	0.0248
0.81	0.0247	0.0250	0.0249	0.0248	0.0248
0.90	0.0245	0.0248	0.0246	0.0250	0.0248
0.99	0.0244	0.0251	0.0249	0.0245	0.0247

Table 1. Simulated worst case FWER of the correlation adjusted two-step procedure with estimated correlation (PI); $K = 2$ experimental groups.

**Max. FWER (K=3)**					
	n				
ρ	5	10	22	50	100
-0.99	0.0248	0.0251	0.0254	0.0249	0.0247
-0.90	0.0252	0.0249	0.0248	0.0246	0.0246
-0.81	0.0255	0.0247	0.0250	0.0251	0.0244
-0.72	0.0254	0.0246	0.0251	0.0250	0.0248
-0.63	0.0256	0.0248	0.0247	0.0250	0.0248
-0.54	0.0256	0.0250	0.0250	0.0251	0.0247
-0.45	0.0253	0.0253	0.0251	0.0249	0.0250
-0.36	0.0251	0.0251	0.0253	0.0252	0.0251
-0.27	0.0247	0.0247	0.0249	0.0250	0.0247
-0.18	0.0240	0.0249	0.0250	0.0249	0.0246
-0.09	0.0236	0.0243	0.0245	0.0249	0.0250
0.00	0.0231	0.0238	0.0244	0.0251	0.0249
0.09	0.0238	0.0247	0.0246	0.0247	0.0249
0.18	0.0240	0.0247	0.0250	0.0250	0.0248
0.27	0.0244	0.0249	0.0247	0.0252	0.0250
0.36	0.0244	0.0250	0.0252	0.0253	0.0252
0.45	0.0251	0.0253	0.0250	0.0250	0.0249
0.54	0.0249	0.0253	0.0252	0.0250	0.0246
0.63	0.0248	0.0252	0.0250	0.0247	0.0249
0.72	0.0250	0.0251	0.0254	0.0250	0.0252
0.81	0.0250	0.0249	0.0248	0.0250	0.0249
0.90	0.0249	0.0251	0.0254	0.0253	0.0251
0.99	0.0250	0.0250	0.0246	0.0249	0.0252

Table 2. Simulated worst case FWER of the correlation adjusted two-step procedure with estimated correlation (PI); $K = 3$ experimental groups.
