# Supplementary figures and images for: Many-to-one comparisons after safety selection in multi-arm clinical trials
Source: PLoS One. 2017 Jun 26;12(6):e0180131. doi: 10.1371/journal.pone.0180131 (PMC5484516; doi:10.1371/journal.pone.0180131)

# Power

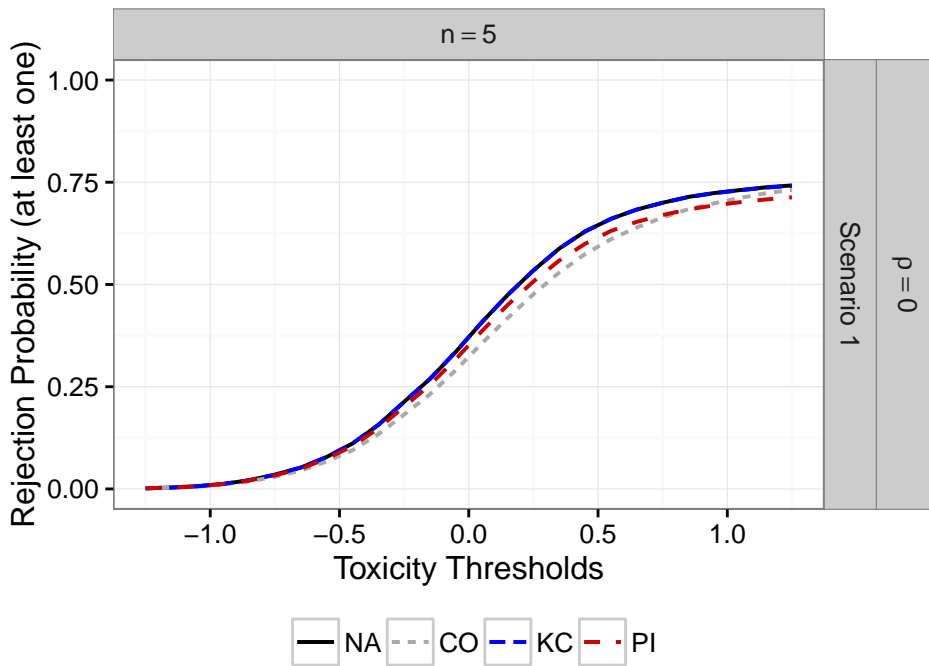

Supplement: S1 Fig — (PDF) [file pone.0180131.s002.pdf]
