# Supplementary material for: Many-to-one comparisons after safety selection in multi-arm clinical trials
Source: PLoS One. 2017 Jun 26;12(6):e0180131. doi: 10.1371/journal.pone.0180131 (PMC5484516; doi:10.1371/journal.pone.0180131)
Supplement: S1 Example — (PDF) [file pone.0180131.s003.pdf]

S1 Numerical Example: Supporting Information for 'Many-to-One Comparisons after Safety Selection in Multi-Arm Clinical Trials'

Numerical example to illustrate the application of the two-step procedure in a clinical trial

The following hypothetical example shows, how to analyse a trial, which has a safety selection step before efficacy testing as proposed.

Let us assume that many-to-one comparisons comparing three experimental arms (e.g., different administration forms of the same substance) to a common control arm shall be conducted with a Dunnett test (DT) for a trial for the treatment of Attention-deficit/hyperactivity disorder (ADHD) using $n_i = n = 22$ patients per arm. For each patient j ($j \in \{1, \cdots, 22\}$) in arm i ($i \in \{0, 1, 2, 3\}$) a score from an ADHD rating scale is calculated at baseline (denoted by $x_{ij}^{(0)}$) and at final visit ($=: x_{ij}^{(1)}$). The primary outcome variable is the difference of this score between these two time-points $x_{ij}^{(0)} - x_{ij}^{(1)}$. The mean difference between baseline and final visit measurements in group i will be denoted as $\bar{x}_i$. For matters of consistency with our framework the measurement at final visit will be subtracted from the baseline value, which implies that higher values of x_i are favorable for the patient. As a continuous safety variable the QT-time is measured at each visit for each patient, since from its pharmacological properties the intake of the experimental substance is suspected to increase the probability for a Long QT syndrome, whereby higher values are considered as less favourable. It is decided to include treatments in the multiple testing procedure for efficacy, that do not have an averse effect on QT-time, i.e., increased values over time. Therefore only groups are forwarded to efficacy testing, if the mean QT-time changes between the final visit minus baseline visit (denoted as $\bar{y}_i$) do not exceed the threshold of $b_i^y = 30$ milliseconds.

According to the definition of the DT, if all groups are selected, group $i \in \{1, 2, 3\}$ is considered to show a significant effect, if the test-statistics D_i exceeds

$b_i^x := t_{\nu=88-3-1,K=3,\Sigma,1-\alpha_{\text{nom}}}$, the $1-\alpha_{\text{nom}}$ quantile of the multivariate t-distribution used for the DT with K dimensions, ν degrees of freedom, and correlation matrix Σ (the correlation matrix of the D_i), which is implicitly defined by the many-to-one comparison problem. The test statistics D_i are defined as

$$D_i := \frac{\bar{x}_i - \bar{x}_0}{\sqrt{V \cdot \left(\frac{1}{n_i} + \frac{1}{n_0}\right)}}, \tag{1}$$

where V is the pooled variance of all selected groups and the control. If less than three groups are selected, then just these groups (and the control) are pooled for variance estimation. Furthermore, the quantile for the multivariate t-distribution is then $b_i^x := t_{\nu=66-2-1,K=2,\Sigma,1-\alpha_{\text{nom}}}$ when two groups are selected and $b_i^x := t_{\nu=44-1-1,1-\alpha_{\text{nom}}}$ when one treatment group is selected. The latter amounts to the conventional t-quantile for comparing a single treatment-control comparison with 42 degrees of freedom).

Now let us assume, that the following results are observed:

	Results			
	Group 0	Group 1	Group 2	Group 3
$\bar{y}_i$ (ms)		46	26	-8
$\bar{x}_i - \bar{x}_0$		2.88	3.84	1.99
V_i	32.22	27.98	28.82	30.52
n_i	22	22	22	22

Table 1. Results of the hypothetical example.

In the following we would like to apply the different two-step selection and testing strategies to these numerical data.

Case 1: No assumption on correlation (CO approach, see section 2) In the planning phase there was no underlying assumption about the correlation between the ADHD score and the QT-Time. The conservative procedure (CO), i.e., always adjusting for the initial number of 3 treatment-control comparison regardless how many treatments pass the safety selection, controls the maximum $FWER_o$ without any assumption related to the correlation. Since in Group 1, the pre-specified threshold of 30 ms is exceeded, just Groups 2 and 3 are selected for the final efficacy evaluation, but the boundaries for the test-statistics D_2 and D_3 correspond to the 3-to-1 case, thus b_i^x ($i \in \{2,3\}$) is set to $t_{\nu=88-3-1,K=3,\Sigma,1-\alpha_{\text{nom}}} = 2.39$. The Dunnett test-statistics take

the values $D_2 = 2.31$ and $D_3 = 1.19$, which follows from the listed values in Table 1, and Eq (1). Since both values do not exceed the critical boundary $b_i^x = 2.39$, no test can be rejected.

Case 2: Assumption of non-negative correlation (NA approach, see section 4.2) Here the design assumption was non-negative correlation between ADHD score and QT-Time across all groups. Therefore it was planned to conduct the two-step procedure using the natural correction (NA). In this case, the selection of just two groups instead of three allows to relax the critical boundaries for the DT: $b_i^x = 2.26$ ($i \in \{2, 3\}$). It is still not possible to declare a statistically significant difference effect of Group 3 compared to the Group 0, since $D_3 = 1.19 < 2.26$, but for Group 2 we obtain a statistically significant result by observing $D_2 = 2.31 > 2.26$.

Case 3: Lower-boundary assumption (KC approach, see section 5.1) Here we consider the same situation as above, but instead of a zero correlation assumption, a lower boundary for the correlation between y and x in all groups is considered to be -0.25, according to data from previous studies. Designing a study using the KC procedure would mean use a lower significance level $\alpha_a < \alpha_{\text{nom}}$ for the quantiles of the multivariate t-distribution above. For $\rho = -0.25$, α_a can be calculated to be equal to 0.0211. The corresponding boundary for the DT for two hypotheses is $b_i^x = 2.34$ ($i \in \{2, 3\}$). In this case, both experimental treatments would fail to demonstrate a statistically significant results with KC assuming a lower boundary of -0.25 for the correlation.

Case 4: Estimation of the correlation (PI approach, see section 5.2) Again we consider the situation from above, but now the correlation between ADHD score and QT-Time will be estimated with the underlying assumption of equal correlations in all groups. In this example, the observed correlations are $\hat{\rho}_1 = -0.10$, $\hat{\rho}_2 = -0.07$, and $\hat{\rho}_3 = -0.17$. Using the Fisher transformation, the best estimate for the true correlation is -0.11. With PI approach the observed correlation is plugged in when adjusting the significance level resulting in an $\alpha_a = 0.0233$. The corresponding DT boundary for two hypotheses is $b_i^x = 2.29$. In this case, Group 2 will yield a statistically significant result.

For methods CO, NA and (KC) used in Case 1, Cases 2 and Case 3, respectively, the critical boundaries b_i^x can be calculated in the planning phase for each potential number of treatments finally being selected. Table 2 gives a summary of these boundaries, depending on the number of selected treatments. In contrast, the calculation of the critical boundaries of the PI approach (Case 4) depends not only on the actual number of selected treatment arms, but also on the observed correlations.

**Critical boundaries for the DT**				
No. of potentially selected treatments:		3	2	1
Case 1	(CO):	2.39	2.39	2.39
Case 2	(NA):	2.39	2.26	2.02
Case 3	(KC), $\rho = -0.25$:	2.47	2.34	2.10

Table 2. Critical boundaries for the DT depending calculated for all potential numbers of selected treatments.
